# Proteomic analysis of three gonad types of swamp eel reveals genes differentially expressed during sex reversal

**DOI:** 10.1038/srep10176

**Published:** 2015-05-18

**Authors:** Yue Sheng, Wei Zhao, Ying Song, Zhigang Li, Majing Luo, Quan Lei, Hanhua Cheng, Rongjia Zhou

**Affiliations:** 1 Department of Genetics, College of Life Sciences, Wuhan University, Wuhan 430072, P. R. China

## Abstract

A variety of mechanisms are engaged in sex determination in vertebrates. The teleost fish swamp eel undergoes sex reversal naturally and is an ideal model for vertebrate sexual development. However, the importance of proteome-wide scanning for gonad reversal was not previously determined. We report a 2-D electrophoresis analysis of three gonad types of proteomes during sex reversal. MS/MS analysis revealed a group of differentially expressed proteins during ovary to ovotestis to testis transformation. Cbx3 is up-regulated during gonad reversal and is likely to have a role in spermatogenesis. Rab37 is down-regulated during the reversal and is mainly associated with oogenesis. Both Cbx3 and Rab37 are linked up in a protein network. These datasets in gonadal proteomes provide a new resource for further studies in gonadal development.

Sexual dimorphism is a common feature in vertebrates. Separation of two sexes promotes the speed of species evolution. The mystery of sex determination has puzzled us for a century. A variety of mechanisms are engaged in sex determination in vertebrates, mainly including genetic sex determination (GSD), in which the sex of offspring is determined by a sex chromosome or key genes; environmental sex determination (ESD) or both GSD and ESD jointly. Male heterogametic XX/XY in mammals and female heterogametic ZZ/ZW in birds are typical GSD systems, while no heteromorphic sex chromosomes exist in many vertebrates, such as the teleost fishes. Moreover, some fish species undergo sex reversal naturally. For instance, male swamp eel (*Monopterus albus*) is transformed from female through an intersex stage^1^,^2^, which is an excellent model system for exploring sexual development.

In mammals, *Sry* is a key sex-determining gene on the Y chromosome^3^,^4^. Mouse contained the *Sry* can develop to male; absence of this gene will develop as female. *Sry* can directly regulate its downstream gene *Sox9* through binding to its gonad-specific enhancer by synergistic action with *Sf1*^5^. Interaction of Sox9 and Sf1 positively regulates the Sertoli cell-specific expression of the *Amh*^6^. Loss *Sox9* impairs sex cord development and Sox8 promotes Sox9 function in testis differentiation^7^. Sex determination is also involved in female determining factors, such as the WNT and FGF pathways. *Fgf9* loss will lead male to female reversal^8^. *Fgf9* represses *Wnt4*, thus promoting male sex determination^9^. Thus, the male determination requires turning on male genes (such as *Sox9*) and off female genes (such as *Wnt4*).

In non-mammals, there is no *Sry* gene and their sex determination mechanisms are complex. Downstream regulatory gene *Dmrt1* (Dsx- and mab-3 related transcription factor 1) in sex determination is evolutionarily conserved, while upstream sex-determining pathways are diverse. The DM domain genes play a similar role in sexual development from fly to worm to mammals^10^. In mice, *Dmrt1* can promote testis differentiation^11^. In birds, Z-linked gene *DMRT1* is required for male sex determination^12^, while W-linked *DM-W* is essential for primary ovary development in *Xenopus*^13^. Sex development in the teleost fish is more complex. Only DM gene that controls sex is *Dmy*/*Dmrt1y* on the Y chromosome in medaka, a duplicated copy of *Dmrt1* on an autosome^14^,^15^. Recently, four novel sex-determining candidate genes have been identified from different fish species, *amhy, Gsdf, Amhr2* and *sdY*^16^,^17^,^18^,^19^. *Amh* and *Amhr* are already known relating to sexual development in mammals, but the *Gsdf* and *sdY* are the new ones first identified in fishes^20^. Together, sex determination and differentiation in fish are complex because of diverse range of species and common molecular mechanisms remain largely unknown.

Taking advantage of the sex reversal characteristic of the swamp eel, we have previously identified six *Dmrt* genes (*Dmrt1, Dmrt2, Dmrt2b, Dmrt3, Dmrt4* and *Dmrt5*); observed that these *Dmrt* genes were up-regulated during gonad reversal^21^,^22^. We have also identified Androgen receptor (*AR*) gene and two *Sox9* genes (*Sox9a1* and *Sox9a2*) in swamp eel, and shown that *AR* is up-regulated during gonadal transformation, has a restricted androgen-dependent transactivation function. Moreover, both Sox9a1/Sox9a2 can interact with AR and regulate AR transactivation^23^,^24^. Despite these advances, mechanistically overall view of sex reversal in this species needs to be studied. Herein, using a comparative proteomics approach, we report a proteome-wide scan for key genes for gonad reversal in swamp eel. 2-D electrophoresis analysis of gonadal proteomes revealed a group of differentially expressed proteins during ovary to ovotestis to testis transformation. One up-regulated gene *Cbx3* and one down-regulated gene *Rab37* during gonad reversal were further analyzed. These data in gonadal proteomes provide new resource for further studies in gonadal development.

## Results

### Identification of the differentially expressed genes in three gonad types

**Figure 1:**
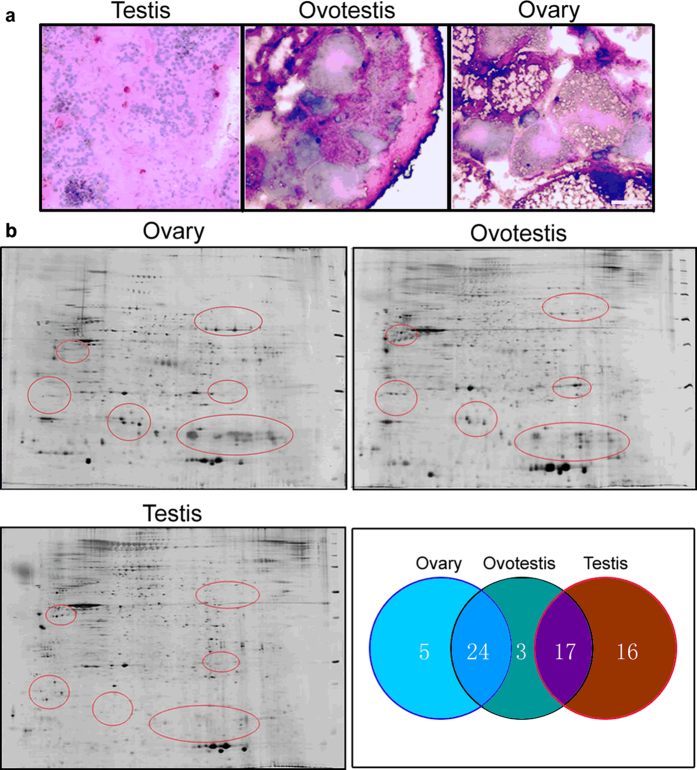
Identification of the differentially expressed proteins among three gonad types. **a**) H.E. staining of ovary, ovotestis and testis tissues. **b**) The 2D protein electrophoresis shows the differentially expressed protein spots among these gonads. The red circles on the 2D gels display the differentially expressed protein spots. Circus plots show the numbers of five kinds of proteins identified: highly expressed in males (16); highly expressed in ovotestis (3); highly expressed in ovary (5); highly expressed in both ovotestis and testis (17); highly expressed in both ovotestis and ovary (24). Detail information of protein spots is showed in Table S1.

To screen the differentially expressed genes during gonad reversal, 2D-electrophoresis was used to isolate the differentially expressed protein spots among ovary, ovotestis and testis. HE staining showed a typic ovotestis structure during the intersex stage, ovary and testis besides (Fig. 1a). Comparative analysis among the 2D gel images of gonad tissues showed many differentially expressed protein spots (Fig. 1b, Fig. S1). There were five spots highly expressed in ovary, three spots in ovotestis, sixteen in testis, twenty-four in both ovary and ovotestis, and seventeen in both testis and ovotestis. These obviously differential spots were subjected to mass spectrum identification. Over 80 differentially expressed proteins were identified (Supplementary Table S1).

**Figure 2:**
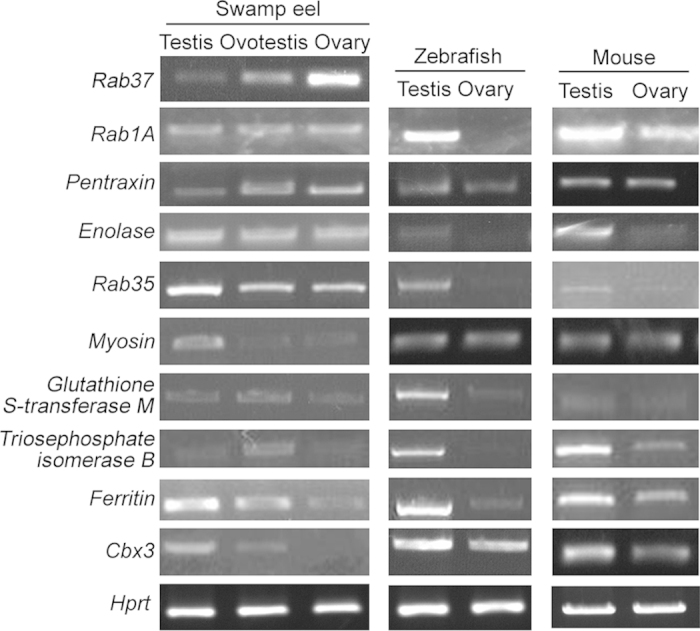
RT-PCR of the differentially expressed genes in gonads of swamp eel, zebrafish and mouse. cDNAs of the differentially expressed genes were identified and non-quantitative RT-PCR was used to examine their expression levels in gonads. Homologs of mouse and zebrafish were also analyzed for comparision. Myosin, Ferritin, Cbx3 are highly expressed in testis; Glutathione S-transferase M, triosephosphate isomerase B are highly expressed in ovotestis. Rab37 and Pentraxin are highly expressed in ovary. Protein expression levels of three spots for each of these genes are listed in Fig. S4. Some of the genes have no difference between gonads, like Rab1A and Enolase. PCR product for each gene has been run under the same experimental conditions and same sizes of gel images have been used for all genes.

To confirm these differentially expressed proteins, we further analyzed ten proteins with high score in MS analysis (Rab37, Rab1A, Pentraxin, Enolase, Rab35, Myosin, Glutathione S-transferase M, Triosephosphate isomerase B, Ferritin and Cbx3). Their corresponding cDNAs were cloned using degenerate PCR and RACE. RT-PCR analysis showed that *Rab35, Myosin, Ferritin* and *Cbx3* are highly expressed in testis tissues of swamp eel, zebrafish and mouse (Fig. 2; Fig. S2). *Rab37* and *Pentraxin* were highly expressed in ovary of swamp eel (Fig. 2; Fig. S2). Tepp was identified in MS analysis of swamp eel testis; unfortunately we did not clone the full-length cDNA from both swamp eel and zebrfaish using degenerate PCR. However, it was specifically expressed in mouse testis three weeks postnatal and located in the nucleus (Fig. S3).

### Cbx3 is up-regulated during gonad reversal

**Figure 3:**
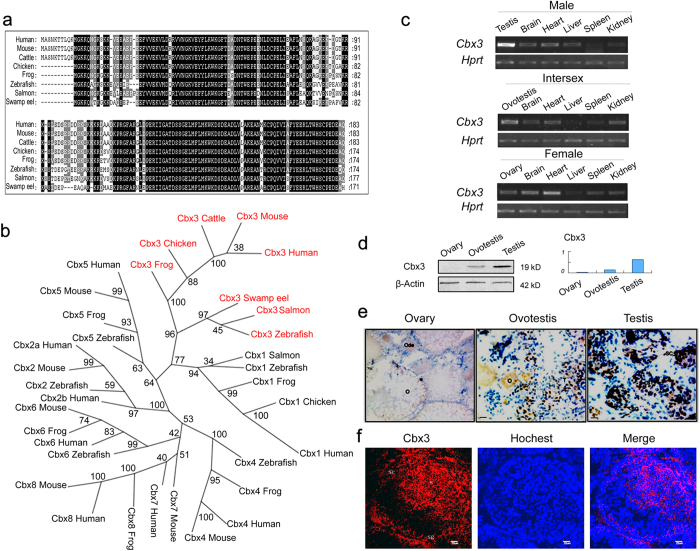
Identification and expression of *Cbx3* gene of swamp eel. **a**). Protein sequence alignments of Cbx3 from human, mouse, cattle, chicken, frog, salmon, zebrafish and swamp eel show an evolutionarily conservation. GenBank access number for swamp eel Cbx3 is KP054389. **b**) Phylogenetic tree depicts the Cbx family from human, mouse, cattle, chicken, frog, salmon, zebrafish and swamp eel. The numbers on the branches represent the boot-strap values from 100 replicates obtained using the maximum-likelihood method. The red color highlighs the Cbx3 cluster. **c**) Non-quantitative RT-PCR analysis in adult tissues from each sex of swamp eel. PCR product for each gene has been run under the same experimental conditions and same sizes of gel images have been used for all genes. **d**) Western blot assay shows Cbx3 expression in three gonad types of swamp eel. The protein gels have been run under the same experimental conditions and same sizes of gel images have been used for all proteins. The right panel shows the intensity values related to Actin. **e**) Immunohistochemistry assay shows that Cbx3 expression in gonads of swamp eel. Marked signals are observed in testis and then in ovotestis. The signals are hardly detected in ovary. O, ovary, Ode, developing ovary. **f**) Immunofluorescence analysis of Cbx3 in mouse testis using anti-Cbx3. Signals are observed in spermatogonia (sg), spermatocytes (sc) and sperm cells (s). The bar, 20 μm.

To investigate expression profile of genes highly expressed in testis, we characterized the *Cbx3* gene of swamp eel. Sequence alignments showed that the Cbx3 was highly conserved from fishes, frogs, chickens to mammals (Fig. 3a). Phylogenetic tree depicted the Cbx family, and *Cbx3* gene of swamp eel was clustered into the vertebrate branch, especially into the teleost fish group (Fig. 3b). RT-PCR analysis of adult tissues in swamp eel showed that *Cbx3* was dominantly expressed in testis, lower in ovotestis and the lowest in ovary (Fig. 3c). The expression was also detected in heart and brain (Fig. 3c). The testis expression of the *Cbx3* was further confirmed at protein level by Western blot analysis (Fig. 3d). Immunohistochemistry indicated that Cbx3 was expressed in spermatogonia, spermatocytes and sperm cells of swamp eel (Fig. 3e). The signals were hardly detected in ovary. Immunofluorescence analysis of Cbx3 in mouse testis further confirmed the expression patterns (Fig. 3f). These results indicated that Cbx3 is up-regulated during gonad reversal and is likely to have a role in spermatogenesis in both fishes and mammals.

### Rab37 is down-regulated during gonad reversal

**Figure 4:**
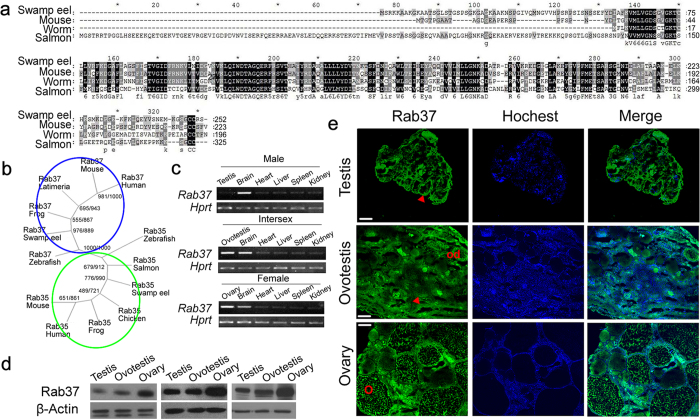
Identification and expression of *Rab37* gene of swamp eel. **a**). Protein sequence alignments of Rab37 of mouse, salmon, swamp eel and worm. GenBank access number for swam eel Rab37 is KP054393. **b**) Phylogenetic tree depicts the Rab37 (blue circle) and Rab35 (green circle) proteins from human, mouse, chicken, frog, latimeria, zebrafish and swamp eel. The numbers on the branches represent the boot-strap values from 1000 replicates obtained using the maximum-likelihood method (first value) and the neighbor-joining method (second value). **c**) RT-PCR analysis of *Rab37* in adult tissues from each sex of swamp eel. PCR product for each gene has been run under the same experimental conditions and same sizes of gel images have been used for all genes. **d**) Western blot analysis shows Rab37 expression in three types of gonads during gonad reversal of swamp eel. Three repeat experiments from each gonad type were performed, which showed a consistent down-regulation trend from ovary through ovotestis to testis . The protein gels have been run under the same experimental conditions and same sizes of gel images have been used for all proteins. **e**) Immunofluorescence analysis in three gonad types. Rab37 is expressed in the gonadal epithelium in testis (red triangle), but not in sperms. In ovotestis, Rab37 is expressed in the epithelium (red triangle) and degraded ovary (od). Rab37 has the most obvious expression in ovary, especially in developing oocytes and mature oocytes (o). The bar, 100 μm.

To explore expression profile of genes highly expressed in ovary, we characterized the *Rab37* gene of swamp eel. *Rab37* was a highly conserved gene in worm, fish and mouse (Fig. 4a). Phylogenetic analysis showed that the eel Rab37 was clustered into the Rab37 branch in vertebrates (Fig. 4b). In adult tissues, *Rab37* was mainly expressed in gonads and brain of swamp eel (Fig. 4c). During gonad reversal, *Rab37* was down-regulated from ovary through ovotestis to testis as indicated using RT-PCR and Western blot analysis, which showed a consistent expression trend (Fig. 4c,d). Immunofluorescence analysis in three gonad types showed that Rab37 was markedly expressed in all stages of follicles in ovary (Fig. 4e). In ovotestis, Rab37 expression was still detected in the degraded ova, while in testis Rab37 had the lowest expression in gonadal epithelium (Fig. 4e). These results suggested that Rab37 is down-regulated during gonad reversal and is mainly associated with oogenesis.

### Cbx3 and Rab37 network

**Figure 5:**
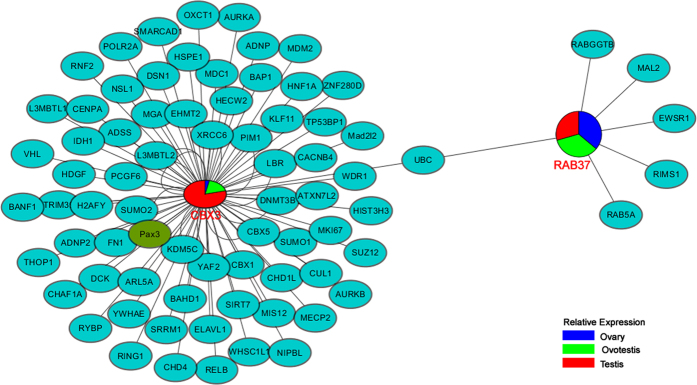
Protein network map of the differentially expressed Rab37 and Cbx3 during gonad reversal. The color of nodes represents expression level relative to Actin among ovary, ovotestis and testis as the color panel indicated. Protein relationships are obtained from BioGrid. All proteins are the swamp eel homologs. Rab37, Cbx3 and Pax3 are identified in the MS analysis.

Because the expressions of Cbx3 and Rab37 are inversely correlated during gonad reversal, their interaction relationship was further investigated. All interactive proteins involved in both Cbx3 and Rab37 were searched in an online interaction repository BioGRID. In the interactive map, Cbx3, Rab37 and Pax3 were identified in the MS analysis. During gonad reversal, Rab37 was down-regulated, while Cbx3 was up-regulated, suggesting their functions in gonad differentiation. Their relationship was further linked up by the UBC protein (polyubiquitin-C), indicating a role of the ubiquitination protein degradation during gonad reversal (Fig. 5).

## Discussion

The teleost fish species is composed of more than 24,000 species accounting for more than half of extant vertebrate species and displays remarkable variation in physiological adaptations, especially in sexual development. Swamp eel is a teleost fish with a natural sex reversal characteristic. Three gonad types (ovary, ovotestis and testis) are obviously detected during sex reversal. In the present study, we have performed a proteome-wide analysis of three gonad types in the swamp eel. The datasets of differentially expressed genes during the sex reversal provide basic data for in depth studies of functions and evolution of sexual development.

We have identified a large group of highly expressed in testis. Cbx3 is a chromotin binding protein and have a conserved function in the formation and maintenance of heterochromatin. Cbx3 can regulate the efficient RNA processing through recruitment of the splicing machinery^25^. Alternative splicing occurs in testis more frequently than in ovary, thus we speculate that Cbx3 has a similar function in RNA processing in gonads, based on its upregulation during gonad reversal. Unlike Cbx3, Tepp is testis specifically expressed, especially in the late stage of testis development, suggesting a role in spermatogenesis.

Of particular interest, we have also detected some genes highly expressed in ovotestis. The genes expressed in ovotestis are likely to have two functions. One is for regulation of the ovary apoptosis and another is to promote testis differentiation. We have identified two genes highly expressed in ovotestis: Glutathione S-trasferase M and Triosephosophate isomerase B. These two genes are all highly expressed in testis in both zebrafish and mouse, suggesting a conserved role in promoting testis differentiation or inhibiting ovary development.

We have also identified a group of genes highly expressed in ovary. *Pentraxin* is a multifunctional gene involved in female fertility, matrix deposition, innate immunity and inflammation^26^,^27^. However, Pentraxin’s function was poorly understood in fish. We showed that the gene is down-regulated during gonad reversal and evolutionarily conserved from fishes to mammals, suggesting a role in ovary development in vertebrates. Rab37 was first identified from the secretory granules in bone marrow mast cells^28^, was localized in various vesicles, such as dense-core vesicles in in PC12 cells^29^, insulin secretory granules^30^ and Weibel-Palade bodies^31^. It regulates TNF-alpha and insulin secretion^32^,^33^. RAB37 also plays an important role in endothelial cell function and embryogenesis in zebrafish^34^ and is involved in tumor cell growth^35^,^36^,^37^,^38^. Here we suggest another function of Rab37 in ovary development. Because *Rab37* is a highly conserved gene from fishes to mammals, we speculate that Rab37 is likely to play a conserved role in follicle development in vertebrates.

During gonad reversal, Cbx3 was up-regulated, while Rab37 was down-regulated, suggesting their respective functions in gonad differentiation from ovary to ovotestis to testis. Notably, both Cbx3 and Rab37 are linked to ubiquitination by the UBC protein (polyubiquitin-C). Indeed, apoptosis and protein degradation during gonad reversal is an important process^39^, which is likely responsible for ovary degradation and spermatogenesis during sex reversal.

There are also some limitations in our 2D results. Due to insufficiency of the fish protein databases, we still have a lot of peptides, which can not match any known proteins. Fish species have their special gene pools including swamp eel. 2D resolution is not precise enough, for example, Rab37 identified from ovary-only spots is expressed highly in ovary and ovotestis, and also lower in testis. There would be also sampling errors as differences of growth and development among individuals of same sex. Thus further verification and functional analysis are needed to completely understand the molecular mechanisms of sex reversal.

## Methods

### Animals

The swamp eels (rice field eels, *Monopterus albus*) were purchased from Wuhan markets in China. Their sexes were identified by microscopic analysis of gonad sections with a crystal microtome (CM1850, Leica, Bensheim, Germany). The animals were treated in accordance with the Guiding Principles for Biomedical Research Involving Animals. The present study was reviewed and approved by the Ethics Committee of Wuhan University.

### Gonad protein preparation

The swamp eels were first washed by ddH_2_O and then disinfected by 75% alcohol on the surface. Each type of sex gonad was collected from three individuals as the triplicate. About 500 mg ~ 1000 mg tissues were collected from one individual and immediately immerged into cold PBS on ice. After identification of the sex type, the samples were cut into small segments, washed by PBS and lysed in lysis buffer (7M Urea, 2M Thiourea, 4% CHAPS) with protease inhibitors added (Roche). After homogenization by a homogenizer (Feiyi, Wuhan, China) (the volume of particles are less than 1 mm^3^), the samples were sonicated by the Sonic Dismembrator (Thermofisher, #FB120220) for 10 min (10 s on, 50 s off, 600 W) on ice. The lysate was centrifuged at 14000 rpm for 20 min at 4°C, transferred the supernatant to a clean tube and normalized to the same concentration (30 mg/ml) by BCA Protein Assay Kit (#23225, Thermo Fisher).

### Two-dimensional differential in-gel electrophoresis

Using the similar method^40^, the IPG strips (Bio-Rad) were rehydrated for 12 h (30 V, 20 °C) with total ~500 μg proteins and then separated by 1D IEF for 500 V for 1 h, 1000 V for 1 h, 8000 V for 10 h in PROTEAN IEF System (Bio-Rad). The strips were then balanced in the buffer (50 mM Tris-HCl pH8.8, 6 M urea, 30% glycerol, 2%SDS, 10 mg/ml DTT, a little bromophenol blue) for 15 min, twice with gently shaking. Proteins were further separated on 12.5% SDS-PAGE gels using PROTEAN II xi Cell (Bio-Rad). Each type of gonads contains three individuals and run three gels separately. After fixed in fixing buffer and stained in Coomassie Blue solution, the gels were washed by H_2_O for several times, then gel images were taken by Image Scanner (Bio-rad) (Fig. S1a).

### Identification of differentially expressed spots

The valid spot analysis was performed using ImageJ (version J2, NIH, Maryland, US) and following the protocols^41^. The intensities of spots were compared among gonad samples, using one gonad type as a control (Fig. S1b). Data were prepared as excel and image files. Microsoft excel software was used to analyze excel files. The statistical significance of image analysis was determined by the Student’s t-test. The chosen spots were picked up from the 2-DE gel and placed into 96-well plates. After washing twice in ddH_2_O, the gels were washed three times in 25 mM NH_4_HCO_3_, and then 50% CH_3_CN for 30 min within rotating. After dehydrating in 100% CH_3_CN and air-dry for 1 h, the samples were suspended in 1.5 uM trypsin (Sigma) with 25 mM NH_4_HCO_3_. After digesting at 37 °C overnight, 96-well plates were centrifuged and supernatant can be used for MS/MS analysis.

### MALDI-TOF/TOF MS, LC-MS/MS analyses and database searching

The digested peptides were analyzed by MALDI-TOF/TOF MS (Bruker-Daltonics AutoFlex TOF-TOF LIFT Mass Spectrometer, Bruker-Daltonics) or LC-ESI-MS/MS (LTQ XL, Thermo Finnigan, San Jose, CA). The proteins were identified by the search program Mascot software ( http://www.matrixscience.com) within the NCBInr database. Two criteria of candidate proteins are indispensable, the protein scores should be more than 60 in MALDI-TOF/TOF MS and at least three peptides matched one same protein in LC-ESI-MS/MS analysis.

### RNA isolated and cDNA synthesis

Total RNAs were extracted from tissues by Trizol reagent from Invitrogen (CA, USA) according to the manufacturer’s protocol. All the total RNAs were digested by RNAse-free DNase I and purified. M-MLV enzyme (Promega, WI, USA) was used to reverse transcribe total RNA into cDNAs with primer CDSIII, 5′-ATTCTAGAGGCCGAGGCGGCCGACATG-d(T)30N_1 N-3′(N = A/G/C/T, N_1 = A/G/C).

### Degenerate PCR

Degenerate primers (Table S2) were designed to amplify the conserved regions based on sequence information of other species, especially the fish species. PCR cycling conditions were: 95 °C for 5 min; 35 cycles, each with 30 s, 95 °C; 30 s, 58 °C; and 30 s, 72 °C; 72 °C for 2 min. PCR products were cloned into pGEM-T-easy clone (Promega) and sequenced.

### Semi-quantitative PCR and RACE

The cDNA templates were prepared from tissues of swamp eel, zebrafish and mouse. Specific primers for swamp eel were designed based on sequencs produced by degenerate PCR. For RACE PCR, the cDNAs were purified by phenol/chloroform (1:1), the 100% alcohol precipitated for 10mins, 70% alcohol washed for one time, and then treated with dCTP using terminal deoxynucleotidyl transferase (TdT) (Promega) for 15 min to add poly C at the terminal. Use the specific primers and CDSIII (for 3′ RACE) or 5MDP (for 5′ RACE, sequence: 5′ GCCACGCGTCGACTAGTACGGGGGGGGGG 3′) to amplify the terminal fragment. PCRs were performed under the conditions: 95 °C, 30 s; 60 °C,30 s; and 72 °C, 30 s for 25-28 cycles (Semi-quantitative PCR) or 35-40 cycles (for RACE). The fragments were cloned into pGEM T-easy (Promega) and sequenced. The detail primers and conditions were listed in Table S2.

### Western blot analysis

The whole extracts from tissues were analyzed by SDS-PAGE and transferred to PVDF membrane (Millipore, MA). The membrane was blocked with 5% non-fat milk powders in TBST (20 mM Tris-HCl pH 7.5, 150 mM NaCl, 0.1% Tween 20) and incubated with the first antibody at 4 °C over night, and then with an AP-labeled or HRP-labeled secondary antibody. The immunoreactive signal was revealed by NBT/BCIP regents or the ECL plus detecting reagents. Anti-β-actin and anti-Cbx3 polyclonal antibodies were purchased from Santa Cruz Biotech (CA, USA). Rab37 antibody was produced by Beijing Protein Innovation (Beijing, China).

### Cell culture and microscopy

COS-7 cells were cultured in DMEM (Cat# SH30022.01B, Hyclone, Beijing, China) with 10% FBS (Cat# SV30087.02, HyClone), 37 °C, 5% CO2. PEGFP-N1 was used to construct a fused protein with Tepp and transfect into COS-7 cells. After 48 h, the cells were fixed with methanol for 20 min at −20 °C and stained with DAPI. Images were taken by a confocal fluorescence microscope (Olympus, FV1000, Tokyo, Japan).

### Immunohistochemistry and immunofluorescent analysis

Tissues were embedded in OCT (optimal cutting temperature) medium (Feiyi, Wuhan China) and frozen at−20 °C, then cut into serial 7 μm sections with a crystal microtome (Leica). For immunohistochemistry or immunofluorescent analysis, sections were permeabilized with 0.1% v/v Triton X-100 for 15 min, washed with PBS for three times, and blocked at room temperature for 30 min in 5% v/v BSA. Slides were incubated at 4 °C overnight in the first antibody. After 5 times of washing with PBS, AP-labeled (CBX3 in swamp eel), FITC (Rab37 in swamp eel) or Cy3 (Cbx3 in mouse) conjugated secondary antibody was applied for 1 h at room temperature. The nuclei were stained with Hoechst33258. Images were taken with a confocal fluorescence microscope (Olympus, FV1000, Tokyo, Japan).

### Phylogenetic analysis

The sequences of the other species were downloaded from NCBI. These sequences were aligned using the ClustalW program. The neighbour-joining (NJ, 1,000 runs) and maximum-likelihood (ML, 100 or 1,000 runs) methods (Phylip, version 3.6) were used to perform phylogenetic analysis.

### Protein network analysis

We used Tblatsn program to search the homologous genes of the swamp eel (E < 0.0001) from human protein database. All human protein sequences were downloaded from Ensembl (GRCh37). The program of Cytoscape^42^ (version 3.1.1) was used to visualize and analyze the interaction network. Gene relationships were obtained from BioGrid^43^ (version 3.2) and Human Protein Reference Database (HPRD)^44^ (release 9) and input into Cytoscape with undirected connection. Finally, the network was displayed using force-directed layout algorithms.

## Author Contributions

R.Z. and H.C. designed the experiments. Y.S., W.Z., Y.S., Z.L., M.L. and Q.L. performed experiments. Y.S. and R.Z. wrote the manuscript. All authors discussed the results and commented on the manuscript.

## Additional Information

**How to cite this article**: Sheng, Y. *et al.* Proteomic analysis of three gonad types of swamp eel reveals genes differentially expressed during sex reversal. *Sci. Rep.* **5**, 10176; doi: 10.1038/srep10176 (2015).

## Supplementary Material

Supporting Information
