# Supplementary material for: Proteomic analysis of three gonad types of swamp eel reveals genes differentially expressed during sex reversal
Source: Sci Rep. 2015 May 18;5:10176. doi: 10.1038/srep10176 (PMC4434955; doi:10.1038/srep10176)
Supplement: Supporting Information [file srep10176-s1.pdf]

Supplementary information for

Proteome-wide analysis of three gonad types of swamp eel reveals genes differentially expressed during sex reversal

Running title: Gonad proteome during sex reversal

Yue Sheng, Wei Zhao, Ying Song, Zhigang Li, Majing Luo, Quan Lei, Hanhua Cheng*, Rongjia Zhou*

Department of Genetics, College of Life Sciences, Wuhan University, Wuhan 430072, P. R. China

*Corresponding authors: Professors Rongjia Zhou and Hanhua Cheng, Department of Genetics, College of Life Sciences, Wuhan University, Wuhan 430072, P. R. China, Fax: 0086-27-68756253, E-mail: rjzhou@whu.edu.cn; hhcheng@whu.edu.cn

Including:

Supplementary Table S1
Supplementary Table S2
Supplementary Figure S1
Supplementary Figure S2
Supplementary Figure S3
Supplementary Figure S4

Table S1. List of the differentially expressed proteins in different gonad types

Female specific

Location of gel	Common name of protein	NCBInr identifier	Protein description	Species	Protein Score	Peptides
V-003-F	Ribosomal protein L19	gi|32034689	A component of the 60S subunit	Actinobacillus pleuropneumoniae serovar 1 str. 4074	43	SNIIKQIEQEQLK<br>LQAFEGVVIAIRNR<br>QNVPSFRPGDTLEVK<br>MSNIIKQIEQEQLK<br>VFQTHSPVVDSISVKR
	Cinorf7 protein	gi|27764002	Cinnamycin biosynthetic gene cluster from Streptomyces cinnamoneus cinnamoneus DSM 40005	Streptomyces cinnamoneus	45	NAEQQSGIR<br>NAEQQSGIRLVAQLTS<br>IQELMVLCSLLPPDGK<br>RIQELMVLCSLLPPDGK<br>IQELMVLCSLLPPDGKLR
V-004-F	Hypothetical protein WS1287	gi|34557651	Hypothetical protein	Wolinella succinogenes DSM 1740	40	RITPIFGSPR<br>MRTLLIGMIWVYQK<br>CNSLFEGGIDYPIISK<br>VWLVPLCPQKDWSAK<br>CNSLFEGGIDYPIISKR
	Polyubiquitin	gi|29169226	Protein stabilization	Camelus dromedarius	70	DQQRLIFAGK<br>GGMQIFVKTLTGK<br>IQDKEGIPPDQQR<br>QLEDGRTLSDYNIQK<br>TLSDYNIQKESTLHLV<br>TLTGKTITLEVEPSDTIENVK
X-001-F	Enolase	gi|72153793	Homodimeric enzymes that catalyse the reversible dehydration of 2-phospho-D-glycerate to phosphoenolpyruvate as part of the glycolytic and gluconeogenesis pathways	Cocos nucifera	65	VNQIGTVTESIK<br>IGSEVYHNLK<br>LGANAILGVSLAASR<br>VKIGLDCASSEFFK<br>SKLGANAILGVSLAASR
A-3-F	Dynein heavy chain	gi|68129568	Germ-line cyst formation，mitosis，oogenesis. Tl	Leishmania major	66	AVLVMAGQLK<br>EQGIEQEIR<br>TLEDNIEKR<br>QRLSELQMK<br>QRLSELQMK<br>FIAQDEPLFK<br>NEVMRVFVDR<br>VEGQLLGTHTGR<br>QLIDLVAEMDK<br>LLTLPNGERIR<br>LLTLPNGERIR<br>QYHGMVALTGAK<br>SPVLFVGESGTAK<br>EEVRVPAPASLK<br>LSWVFEQIRR<br>LVMPTNALSMVK<br>CLFFVRVDHR<br>DLSRIYEGLCR<br>EMAERQLQACR<br>MVQSELEFFLR<br>GSSDLSEESVLMR<br>HWTELMRVTDK<br>LIVFVDDINMPK<br>DKVMLSFQMAVR<br>SISLGQGQGEEAMR
A-5-F	GA18684-PA	gi|125979875	Cholesterol binding，electron transport，steroid biosynthetic process	Drosophila pseudoobscura	65	SYRFEPIGER<br>RATELVNTHLK<br>RATELVNTHLK<br>SVMAHLLRSYR<br>TPEGASLSVKEIR<br>QHQFNLDEVNR<br>TLTPAFNYTMIK<br>GNDVVISPMYMGR<br>IEATTFIPFMTGAR<br>IEATTFIPFMTGAR<br>RATANCQINDFFIPK<br>YAMVMIKSVMAHLLR<br>FVMYNMTLHTDHQQQCR<br>FVMYNMTLHTDHQQQCR<br>IEATTFIPFMTGARSCLGQR
	Putative ATPase	gi|68550161	ATP synthase gamma subunit	Pelodictyon phaeoclathratiforme BU-1	58	ALMLDIGLMR<br>FESLALVDLER<br>FESLALVDLER<br>AFDALCLAQVAR<br>RYFFIGGMPAAVK<br>KAFDALCLAQVAR<br>KAFDALCLAQVAR<br>YLSGMPNDIEYAK<br>SSSAEVDYLAVLDGK<br>VTRICSDLEVLLQQK<br>FCLDSVFTSLSQHIGK<br>VDRFCLDSVFTSLSQHIGK
B-0-F	BAPKO_0539	gi|111115340	Hypothetical protein	Borrelia afzelii PKo	69	ISNKESILK<br>AIDMHGSWK<br>LEKLWETLK<br>TNKENLESIK<br>SVFMLEDLNK<br>TGDIESLVIEK<br>DNLVEYKSDLR<br>MESVQVGAIDLMK<br>TIDSDIMQAKER<br>VNLEDFIEDKTK<br>AEEIFSNLQSEAK<br>EIENSFGFYEDK<br>SDNQKQIDDFLDR<br>SLVESYDEMQIYK<br>INSLEESIRIEMGK<br>NNSIFLEAYSLKDK<br>FGEITNDLKNNIESK<br>EMIFELESQLKNLK<br>IAEMDHNFEIIEQR<br>DNVYSRINSLEESIR<br>SLVESYDEMQIYKAK<br>DMESEIALMETNITGR<br>LNSYITTLSEEFKHSNK<br>LDLEFNDFLEKVEFNIVK<br>HSTLEIESVKNDLQEQIDK
D-6-F	Hypothetical protein FP2506_06446	gi|114704552	Hypothetical protein	Fulvimarina pelagi HTCC2506	57	WTFEPLGEK<br>IDVKYVDGPFR<br>TLLVADMTVAYK<br>DNKTLLVADMTVAYK<br>WTFEPLGEKQCDVR<br>QCDVRFFIDYEFK<br>TLSMLMGSMFDYAFR

	IscU protein	gi|78186124	Iron-sulfur template protein	Pelodictyon luteolum DSM 273	56	DEAESLLEK<br>TKLGTVCGQCK<br>DEAESLLEKFK<br>IHCSVLGDKALR<br>GMTLTEAFNVSPK<br>MLQAGEWAYSEKLK<br>AAINNYYVRNGMEDR<br>GMTLTEAFNVSPKDVAK
D-7-F	Hypothetical protein	gi|114675515	Similar to zinc finger protein 709, regulation of transcription, DNA-dependent	Pan troglodytes	64	AFSFPSSFR<br>HMRSHTEHR<br>AGNARQEFPDK<br>HMAVQRGDGPYK<br>IHTGEKPYECK<br>CGKAFSFPSSFR<br>HMIRHTGNGPHK<br>HMIMHTGNGPYK<br>QCGKALSCPTSFR<br>ECGKAFISLPSYR<br>AFSCSSSVRMHER<br>AFGHYDNLKVHER<br>ECGKAFASLNSLHR<br>AFNYHNSLQTHER<br>AFFWPSLLHMHER<br>THTGEKPYECKTCR<br>THTGENPYECKECGK<br>SYECQQCGKAFTHSR
E-0-F	Similar to pentraxin	gi|125854012	The prototype of the long pentraxin family: it shares similarities with the classical, short pentraxins but differs for the presence of an unrelated long N-terminal domain<br>Oocytes ovulated by ptx3-deficient mice can be fertilized in vitro, indicating that the oocyte develops normally in the absence of PTX3 and that fertilization failure observed in vivo is actually a result of the defective cumulus expansion.<br>SAP was detected on ejaculated spermatozoa, in seminal plasma and in tissue sections from the male reproductive tract.	Danio rerio	63	VATELQGER
F-1-F	Novel protein similar to vertebrate keratin type I family	gi|94732967	Type I keratins (or Type I cytokeratins) constitutes the Type I intermediate filaments (IFs) of the intracytoplasmatic cytoskeleton, which is present in all mammalian epithelial cells. Most of the type I cytokeratins consist of acidic, low molecular weight proteins which in vivo are arranged in pairs of heterotypic Type I and Type II keratin chains, coexpressed during differentiation of simple and stratified epithelial tissues.	Danio rerio	63	NHAEELAALR<br>KNHAEELAALR<br>SDLEMQIEGLK<br>QQYEGITEKNK
H-1-F	Similar to golgi autoantigen, golgin subfamily a, 4	gi|109484079	The golgins are a family of proteins, of which the protein encoded by this gene is a member, that are localized to the Golgi. This protein has been postulated to play a role in Rab6-regulated membrane-tethering events in the Golgi apparatus	Rattus norvegicus	81	VSSLEAELR<br>EAAPSQSEVR<br>MKELHMAEK<br>LRLQHELSR<br>VLFEYMMGR<br>RYEEILDAR<br>EQCALLLSEK<br>EELQMDQQAK<br>LAVHLEAEKSK<br>KECDLETELK<br>MKVEMDEQIK<br>DQHEKLIEEK<br>EHQQELDILR<br>RPEVPASSTGRR<br>QALEAELQEQR<br>ELDDVNSSVKSR<br>QQIESLTGAHQR<br>ECEQEAEEKLK<br>QALEAELQEQRR<br>QLLSQMEAKVQQCAK<br>EQELREQVHNLEDR<br>EKMSALEQVDHWSNK<br>YSELVTAFQTLQREK<br>EESSPSQSGDTQTFAQK<br>LHAEQLASKEQELSQK
	FOG: TPR repeat (ISS)	gi|116000697	Increased serum survival protein; involved in complement resistance	Ostreococcus tauri	60	AETAAEAARAR<br>IADAREDVVR<br>SMCWLKLAR<br>GQKGGSMESSTR<br>LARWSDAEADCDAAAR<br>GGSMESSTRAETAAEAAR<br>SESATALANRSMCWLK<br>WISDANAEDDALRAASR
H-2-F	OSIGBa0145N07.7	gi|116309371	Oryza sativa indica (guangluai4) genomic DNA, chromosome 4, BAC clone: OSIGBa0145N07	Oryza sativa (indica cultivar-group)	60	KPNVAPPQQQK<br>GIPMVAGPASQSK<br>AAAEQIAHEDAR<br>ATATDHGAAPQPLR<br>AAAEQIAHEDARK<br>GIPMVAGPASQSKLK<br>LAMQQTSTILAPPMTAK<br>QKQQGIGFAGAVAPPPSPR
H-3-F	RAB37	gi|119609588	Member RAS oncogene family, involved in transp	Homo sapiens	54	TILVGDSGVGK<br>ADMSSERVIR<br>TSLLVQFDQGK<br>LQIWDTAGQER<br>EYGVPFLETSAK<br>TGMNVELAFLAIAK<br>AWLTEIHEYAQR
V-001-F	Hypothetical protein RPA2489	gi|39935555	Hypothetical protein	Rhodopseudomonas palustris CGA009	53	LLRPPEPEPVADLQK<br>ASHRLLCLWVMWSR<br>LLCLWVMWSRVPAGR<br>TRTAFPFATQDVSALAR<br>LLCLWVMWSRVPAGR<br>LGHVEMLNLLARSAGYR<br>MTRTAFPFATQDVSALAR<br>LLRPPEPEPVADLQKVAQATR
V-002-F	Hypothetical protein YintA_01003639	gi|77976539	Hypothetical protein	Yersinia intermedia ATCC 29909	54	KELIAQLYK<br>IVWFDPANAVK<br>FVHRIVWFDPANAVK<br>SKNAGPYELVLDILFK<br>NAGPYELVLDILFKDK<br>IVWFDPANAVKVVMPR
	ISPpu15	gi|126667756	Transposase, binds to the end of transposon to another part of the genome by a cut and paste mechanism or a replicative transposition.	Marinobacter sp. ELB17	52	IRQELSVPR<br>EATKAAKPQGK<br>MEATKAAKPQGK<br>TWLEANAGKVAK<br>TLKTWLEANAGK<br>NYLHPIIMQGSSEHKR<br>GDLQISNVLAENAIRPFAVGR

	Hypothetical protein Rsph17025DRAFT_3660	gi|83370167	Hypothetical protein	Rhodobacter sphaeroides ATCC 17025	51	GLGAAVANLRR<br>IGRGASTAEVR<br>YPLAETVLARAA<br>IAAPLHDLRIGR<br>GASTAEVRATLAELR<br>LDNPRLVVILLDWSR<br>MLSTIDGHTPSVARAVR
**Male specific**						
r-1-m	Hypothetical protein Tb927.2.5220	gi|84043808	Hypothetical protein	Trypanosoma brucei TREU927	57	CEPIVVAMR<br>QWFLTIDRK<br>ELHEATENELR<br>KLGNGVCGLSSASDK<br>AMHEGYNEEVRHR<br>AMAAQLENCVRLIR<br>SGMSSDTRLPAAVLER<br>EELAEFRQWFLTIDR<br>NVLEMGTGDRALMWDVQQR<br>NVLEMGTGDRALMWDVQQR<br>VINYSHSTSRAMAAQLENCVR
	PREDICTED: similar to MAWD binding protein	gi|73953277	Unknown protein 32 from 2D-page of liver tissue	Canis familiaris	55	KDMHAFQCSR<br>MGESEREGGGGAR<br>DATWFGGQMRR<br>ATETCRPALARPER<br>IAREMNLSETAFIR<br>GEPGGQTQTFDFYSR<br>KLNPTDNFTQSSCFGLR<br>GNPAAVCLLENKLDEDVHQK<br>SFLENLKVNTQDLLQVENTGK
E-5-m	Tepp	gi|38637667	Specifically expressed in testis, prostate, and plac	Xenopus laevis	69	SQERYGYR<br>MEMDSVVHK<br>MEMDSVVHK<br>MEMDSVVHK<br>FTSAAADWSQR<br>NARFTSVAAPDHR<br>SAGNLCSLNSSIHPWR<br>SAGNLCSLNSSIHPWR<br>GYAVCFLPTDGSRSWK<br>YGYRSGGCWTAPAPTSSR
E-6-m	Chain G, Diol Dehydratase-Cyanocobalamin Complex	gi|6980838	Diol Dehydratase-Cyanocobalamin Complex From Klebsiella Oxytoca	Klebsiella Oxytoca	52	MNTDAIESMVR<br>HPEWVKTATNK<br>SARVSDYPLANK<br>EELLAIADDLESR<br>VSDYPLANKHPEWVK<br>MNSLQGEAPAAAPAAGGASR
	DPY30 domain containing 1	gi|55959187	Dpy-30 motif. This motif is found in a wide variety	Homo sapiens	52	MESIYLQK<br>MNMENLVR<br>ENVTMEQLR<br>TLAEISDRYGAP<br>ENVTMEQLRQK<br>ELALMEQEMMER<br>ELALMEQEMMERLK<br>ERELALMEQEMMER
	Chromobox protein, partial	gi|4585819	Similar to chromobox protein 3, chromosome bingding protein	Oryzias latipes	52	WKDSDEADLVPAR<br>CPQVVISFYEER
	RAB35	gi|50418486	Member RAS oncogene family, related to AR and	Danio rerio	55	VEEMFNCITE
E-8-m	Putative transient receptor protein 2	gi|37779086	Similar to myosin, light chain 9. Myosin, a structural component of muscle, consists of two heavy chains and four light chains. The protein encoded by this gene is a myosin light chain that may regulate muscle contraction by modulating the ATPase activity of myosin heads. The encoded protein binds calcium and is activated by myosin light chain kinase. Two transcript variants encoding different isoforms have been found for this gene.	Pagrus major	100	GNFNYAEFTR<br>LNGTDPEDVIR<br>EAFNMIDQNR<br>EDLHDMLASLGK<br>KGNFNYAEFTR<br>FTDEEVDELFR<br>DGFIDKEDLHDMLASLGK<br>ATSNVFAMFDQSQIQEFK<br>NAFACFDEEGSGVIHEDHLR
P-1-m	Hypothetical protein OsI_002613	gi|125526652	Hypothetical protein	Oryza sativa (indica cultivar-group)	58	TVATIDWSMK<br>TRPANSITVFDVDK<br>ACATARNASNTMLEVR<br>YQLSWRNAVEDITSK<br>LQYYTSSMVDDVFTR<br>LGEDIKVKPWAHTVYGK<br>TRPANSITVFDVDKACATAR<br>TPRLQYYTSSMVDDVFTR
	Hypothetical protein	gi|145481747	Hypothetical protein	Paramecium tetraurelia	58	QALQYQNNKK<br>ELLSNSVGEAIK<br>GFLPNQFRSIVK<br>QSYLQSNIFHDTK<br>MLLFPESRYPIENR<br>HPGKTNFMASDLSFLK<br>QEQEINVKLNQIEIPK<br>QSQQSVLFNTLGKYYEEHK
P-3-m	Unnamed protein product	gi|116056202	Hypothetical protein	Ostreococcus tauri	56	VAMQDALTR<br>FNARGEPVR<br>DFTYHLLDTR<br>HVTSAHAGATNDK<br>FVIRGDDFHTR<br>FFDEGDDGDLHELSAK<br>DFTYHLLDTRDGVQK<br>HVTSAHAGATNDKFVIR<br>MHVTSAHAGATNDKFVIR
P-4-m	Landform specific protein	gi|929819	Involved in the induction of the landform of the liverwort Riccia fluitans L	Riccia fluitans	43	ATQAWESTK<br>DYAVDTAVQGK<br>TEQAKQYGAEK<br>QYGAEKTEQAK<br>TEQAKGAASETAGTAQDK<br>QTTSDTTTTAGNKAVEAK<br>DYTGEKAGQAGQYTTDR
q-1-m	Keratin 10	gi|87045985	Involved in epidermis development, keratinocyte	Oryctolagus cuniculus	56	SEITELRR<br>KNHEEEMK<br>LAADDFRMK<br>VTMQNLNDR<br>QSVEADINGLR<br>MKYENEVALR<br>SQYEQLAEQNR<br>QSLEASLAETEGR<br>SQYEQLAEQNRK
q-3-m	ZFP37	gi|122938222	Involved in cell differentiation，germ cell development	Lemur catta	52	AFTYSSSLTK<br>AFGHSSSLTYHMR<br>THTGEIPYECNECGK<br>IHTGEKPFECNECGK<br>VHTGEKPYDCKECGK<br>SHTGEKPYECKECGK
**Intersex**						

U-005-I	Hypothetical protein Pcal_1914	gi|126460518	Hypothetical protein	Pyrobaculum calidifontis JCM 11548	58	ALFSDTQWLGK<br>MLTTRLATPPSK<br>FYRDNGLQNPASDPAK<br>EYVPALVEMLKMAASK<br>GLSYFKDVGCVACHSIK<br>FLTQPPRDATTMTAQIAAYK<br>SLYGDQWDKEYVPALVEMLK
U-006-I	5-formyltetrahydrofolate cyclo-ligase	gi|121538171	An enzyme catalyze chemical reaction	Anaeromyxobacter sp.Fw109-5	65	LERSHAIAQR<br>LAFARCAPEALVR<br>GPMGALEPPAEAPPVPR<br>GGGYYDATLEAMPRAAR<br>SHAIAQRLEEVPCFR<br>LGRGGGYYDATLEAMPR<br>MPGEAILSATGLTPVEEK<br>DAVDCVLMPGVAFSPDGHRLGR
W-001-I	Hypothetical protein RPB_3518	gi|86750628	Hypothetical protein	Rhodopseudomonas palustris HaA2	51	VVLQLALDR<br>ADFTRGNLTPR<br>DGRSLISHHQFAAGER<br>LARHYGLAPPSGTARPK<br>VTIDDGESPLAWLARR<br>GNLTPRVTSHWGAPTGR<br>AEIDAFRAQHLSLAER
	Hypothetical protein CC1G_00500	gi|116509386	Similar to GDP dissociation inhibitor 2(100%),GDP dissociation inhibitors are proteins that regulate the GDP-GTP exchange reaction of members of the rab family, small GTP-binding proteins of the ras superfamily	Coprinopsis cinerea okayama7#130	49	VERVQTLHK<br>DGIAALKELLK<br>AVPVTSIVLVTDR<br>LTYNMILNLLR<br>DGHFASGALAILLK<br>LRAFEFQETLNLR<br>SYFVLALVDPETKAGR<br>TVKVDTTAIVDTHLIAK<br>KNQTLLLLGMSKPQGAK<br>ELLKEWVPSGVVPEVDWSR
	Acyltransferase	gi|125632053	An enzyme involved in beta oxidation	Cupriavidus sp. MApud10.1	48	IPFAFGTVR<br>AMYPVLQELR<br>HGLGTIIEGIRVR<br>VRSTLSNVHLDLPSTR<br>ATYGPTGPFNAKPFSGGR<br>AMYPVLQELRIPFAFGTVR
	Hypothetical protein CHY_1937	gi|78044628	Hypothetical protein	Carboxydothermus hydrogenoformans Z-2901	56	NNEVVVIIETEK<br>VALAGPLSDDIAKK<br>ALLPLGFTESKVR<br>HVEKWNPHTER<br>VNEAANILADFGGR<br>GLFKALLPLGFTESK<br>EVIIAYFKSLEQTER
W-002-I	Ribosomal protein L15	gi|94312229	Ribosomes, the organelles that catalyze protein synthesis, consist of a small 40S subunit and a large 60S subunit.RPL15 are high expressed in some esophageal tumors.	Ralstonia metallidurans CH34	52	LEAAEVDLLVLK<br>MQLNNLKPADGSK<br>QLNNLKPADGSKHAK<br>MQLNNLKPADGSKHAK<br>DIERLEAAEVDLLVLK<br>GLGATAGAKAAIEAAGGQIEA
	Transcription elongation factor	gi|145571454	Involved in transcription elongation	Pseudomonas stutzeri A1501	50	LHEIETALAAYR<br>GLEAAYLADGQRR<br>LQHDGREILVITAR<br>VRVGALLCLDHDGAER<br>YDTRGLEAAYLADGQR<br>EILVITARSPLGQGLLGR
	Hypothetical protein slr0397	gi|16331121	Hypothetical protein	Synechocystis sp. PCC6803	46	KFTSAWQAYGEK<br>RLWGVLATMVSPA<br>SVSLVEQTKEQGK<br>YPIPAVAPGQLDVEGIR<br>KYPIPAVAPGQLDVEGIR<br>LLSLLTELFFFSSQDGPRR
B-1-I	Calcineurin-like phosphoesterase	gi|124268885	Enzyme	Methylibium petroleiphilum PM1	51	EQHLRTDK<br>AAGYFMHVMR<br>CDGRLFDAAAVR<br>WCDFDLFGAAGR<br>AMRAAGYFMHVMR<br>LFDAAAVRDEALACR<br>HDYSVEHPPGMPRAR<br>WPVPVLFVAGNHEFDRR
	Disulfide interchange protein (DsbC)	gi|16121196	Interchange Reactions Between Cystamine And Cystine Peptides	Yersinia pestis CO92	57	SIWCMADR<br>NKAFDDAMK<br>QGLSSQAEKDMR<br>LEALSSEMIVYK<br>NNDISPATCKTDISK<br>EMLQMLNAHQASLKAGG<br>HVITVFTDITCGYCR<br>LHEQMKDYNALGITVR
B-5-I	Unnamed protein product	gi|829149	Similar to zinc finger protein 250, novel genes associated with cell proliferation	Homo sapiens	48	STLMNHER<br>AFNHSTVLR<br>RTLLQHQR<br>VHTGEKPHR<br>VSSDLAQHHK<br>IHTGEKPYTCSECGK<br>VHTGEKPHRCNECGK
B-2-I	Glutathione S-transferase M	gi|47086689	Belongs to the mu class, functions include the detoxification of electrophilic compounds, including carcinogens, therapeutic drugs, environmental toxins and products of oxidative stress.	Danio rerio	316	IVQSNAIMR<br>LLLEYTGTK<br>QFSDFLGDR<br>KIVQSNAIMR<br>QFSDFLGDRK<br>VDILENQAMDFR<br>KHNLCGETEEEQMR
**Female and intersex**						
B-8-I	Triose-phosphate isomerase B	gi|126211567	An enzyme that catalyzes the reversible interconversion of the triose phosphate isomers dihydroxyacetone phosphate and D-glyceraldehyde 3-phosphate.	Poecilia reticulata	61	HVFGESDELIGQK<br>LDAKFGVAAQNCYK<br>RHVFGESDELIGQK<br>DCGVNWVILGHSER<br>TASPQQAQEVHEKLR
E-1-F	Putative NBS-LRR resistance protein	gi|118140604	Putative protein	Rosa hybridcultivar	62	TLGSLLYNK<br>YAGGHPLALK<br>DWFGLRSR<br>LQNTPDKTVF<br>TLGSLLYNKR<br>LVGEKDWFGLR<br>FPNTTHGLVDLQK<br>HSMSFARYAGGHPLALK
	Hypothetical protein TVAG_211660	gi|123344281	Hypothetical protein	Trichomonas vaginalis G3	68	VLFVNDMR<br>NPELMISVR<br>SNIEFETVR<br>IGDLNVRYSK<br>VKVLFVNDMR<br>IGRTYNITQR<br>SNIEFETVRK<br>ETFEVDEIDK<br>VKVLFVNDMR<br>TNHWVRNASTQK<br>VLFVNDMRAAETELLEK

	Putative guanine nucleotide-binding protein Rab1A	gi|37625107	Member RAS oncogene family, involved in several transport steps of the cellular trafficking pathway.	Aeromonas hydrophila	62	INFSDRLK<br>RPMLDQVK<br>STQVSIDEGER<br>YVIGAMAPVSSK<br>DSDAIKYVIGAMAPVSSK<br>VANTLVSMLETDRINTEFR
	Valyl tRNA synthetase	gi|62321074	Enzyme for valyl tRNA	Arabidopsis thaliana	61	EDNANKLAK<br>LPAPKDTER<br>VESEMDTVLATVK<br>MPFVTEELWQR<br>PFVTEELWQRLPAPK<br>MMSVSTYEEKVPANIK<br>VESEMDTVLATVKCMR<br>SHELEIRTLANLSSLEVVSK
E-2-F	Putative NBS-LRR resistance protein	gi|118140604	Putative protein	Rosa hybridcultivar	74	TLGSLLYNK<br>YAGGHPLALK<br>DWFGLRSR<br>LQNTPDKTVF<br>TLGSLLYNKR<br>LVGEKDWFGLR<br>FPNTTHGLVDLQK<br>HSMSFARYAGGHPLALK<br>NQLVLVTHGVEKPYELK
E-3-F	Chorismate mutase	gi|28198342	Involved in responsing to biotic stimulus, biosynthetic process	Xylella fastidiosa Temecula1	65	MSLSSLLR<br>ETAVLQSVR<br>TRPNLIALR<br>QLNLDNLHHLALIR<br>ETAVLQSVRDQAPQYR<br>LDPDLAARFFSAQIESNK
	Hypothetical protein TVAG_422430	gi|123414362	Hypothetical protein	Trichomonas vaginalis G3	62	RNSAVPNIR<br>YQRAVYLGGTVVK<br>IAVRPTDRISGEDITR<br>SYSVAPVSNWDGPVTVDEMR
	Two component Transcriptional regulator, Winged helix family	gi|115377047	Transcriptional regulator	Stigmatella aurantiaca DW4/3-1	62	EQAYIKTVR<br>DDAREQAYIK<br>TTLAADGREMR<br>LLRVFLDHPQR<br>MLPPNLQVTEAGR<br>MVGEYLQRNGLR<br>NVPVVMLTARDEETDR<br>SEGYVFCQPVILLGESA<br>TVRSEGYVFCQPVILLGESA
E-4-F	Hypothetical protein lpl0234	gi|54293188	Hypothetical protein	Legionella pneumophilastr. Lens	61	AEKQHAHR<br>EITNTLFR<br>VMILELLDAK<br>EITNTLFRLDK<br>LALSQSNYGWRDLIR<br>YDSTEPGLQKLPPEIK<br>LPPEIKVMILELLDAK<br>HLPECTKEITNTLFR
	Spectrin beta 3	gi|55926127	Form a scaffolding and play an important role in maintenance of plasma membrane integrity and cytoskeletal structure	Mus musculus	57	RHEAFQK<br>GERQTLPR<br>VYTPREGR<br>LQKAYAGDK<br>DWLDKVDK<br>GEMSGRLGPLK<br>SLDDFQAWLGR<br>WQQFRSLADGK<br>LYASLKELAGER<br>KNQEAVQQLLGR<br>VLDHAMEAEHLVEK<br>VPTLEQHYEELQAR<br>VDSANALANGLIAGGHAAR<br>AASAGVPYHGEVPVSLAR<br>AMTMPPVSQPEGSIVLR<br>LGEVQAGWEDLRATMR<br>LLEVLSGETLPKPTKGR<br>SIITYVATYYHYFSKMK<br>LEALGTGWEELGRMWESR<br>QLAASSQDMIDHEHPESTR<br>LWIDEKMLTAQDVSYDEAR<br>GPAPSPMPQSRSSEAAHGATLPTR
C-1-F	Unnamed protein product	gi|47225287	Unnamed protein	Tetraodon nigroviridis	143	KAILFCLSK<br>YALYDATYETK<br>HEWQVNGLEDLK<br>VTDEVIAVFNDMK<br>YALYDATYETKETK<br>HEWQVNGLEDLKDR<br>VTDEVIAVFNDMKVR
	Hypothetical protein TVAG_422430	gi|123414362	Hypothetical protein	Trichomonas vaginalis G3	47	IAVRPTDRISGEDITR<br>SYSVAPVSNWDGPVTVDEMR<br>AAVEGEWSSGR<br>NAGNAGIPASMR<br>EPVHVDTNFK<br>QNADLEAQHER<br>TETGPDGKMLCPK<br>CGEQEMKTIQVGK<br>YVPCPVCTQLMNRVNFAR
C-5-F	Polyprotein	gi|3136204	Virus protein	Sindbis virus	50	NFTVDRDGL<br>TVRNFTVDR<br>WVFNSPDSIR<br>QCVAYKSDQTK<br>SDQTKWVFNSPDSIR<br>TGTVTTRTEITGCTAIK<br>SWGYRPWR<br>AYEYTAMRR<br>DMRFYGPQR<br>MPKPTDAFASR<br>LKAYEYTAMR<br>RSWGYRPWR<br>DDPGEDQPPDAR<br>AQAQAEEAEELFR<br>SRQLGLCDDYEAR<br>MAECLARSHLQPTPR<br>VTEGEEASQAAGSQHRR
C-6-F	Paired-box 3 protein	gi|20330101	This gene is a member of the paired box (PAX) family of transcription factors. Members of the PAX family typically contain a paired box domain and a paired-type homeodomain. These genes play critical roles during fetal development.	Canis familiaris	56	MTTMAGAVPR<br>QVTTPDVEKK<br>GEEEEADLER<br>RENPGMFSWEIR<br>SGFPLEVSTPLGQGR<br>ENPGMFSWEIRDK<br>YQETGSIRPGAIGGSKPK<br>MTTMAGAVPRMMRPGPGK

	Hypothetical protein	gi|94397007	Similar to Spetex-2E protein	Mus musculus	54	ENHFYCFR<br>MPQDISEALSKCK<br>EQIALEECNIETK<br>LEMQHDQVMTDLK<br>MLEMQHDQVMTDLK<br>LEMQHDQVMTDLKR<br>MLEMQHDQVMTDLKR
C-7-F	Hypothetical protein An02g12390	gi|145233989	Similar to coactivator bridging factor 1 (Mbf1), nervous system development	Aspergillus niger	58	MTQKELATK<br>GTGIGQEKFPK<br>IGARHTGGPATR<br>NEDGYKMTQK<br>SDWDSVTRIGAR<br>MSDWDSVTRIGAR<br>CNTTITVVQDFER<br>GTAAPDQKVLSAMER
	Mst3 and SOK1-related kinase	gi|89272825	The protein kinase localizes to the Golgi apparatus and is specifically activated by binding to the Golgi matrix protein GM130. It is also cleaved by caspase-3 in vitro, and may function in the apoptotic pathway	Xenopus tropicalis	56	ADPEELFTK<br>IGKGSFGEVFK<br>GLDYLHSENK<br>YYGSYLKGTK<br>LADFGVAGQLTDT
	Predicted protein	gi|145340503	Predicted protein	Ostreococcus lucimarinus CCE9901	55	CKHDWMR<br>VKAGCAGDEAK<br>TYEFVSRSR<br>DGVEVLMLNSK<br>CVAHVFTMEVK<br>DGVEVLMLNSKK<br>GGWELDETASEAAAR<br>CEYETWPEEGERTR
C-8-F	Ferredoxin	gi|58040345	Iron-sulfur proteins that mediate electron transfer in a range of metabolic reactions	Gluconobacter oxydans 621H	55	AAMLSEAPHK<br>AAMLSEAPHKD<br>TYVVTENCIR<br>YSTQWPNMTR<br>MTYVVTENCIRCK<br>IDAMPDAEEWKDKPGK
	Hypothetical protein Tfu_1767	gi|72162168	Hypothetical protein	Thermobifida fusca YX	52	DEQHTSATPR<br>DDSEAIVEVAK<br>DLPPEARGNEK<br>MDEQHTSATPR<br>DDSEAIVEVAKFDDGYR<br>WHDTSNVVWMRSSLSR
	Putative secreted glycine and proline rich salivary protein	gi|67083232	Putative protein	Ixodes scapularis	50	GSSQPDELPR<br>SEMPTTPSGDK<br>SEMPTTPSGDKSK<br>SPPGLPTTESTPHTGK<br>RSPPGLPTTESTPHTGK<br>SDLSAPTTNSTKSSASVR<br>DIRMGMMTVPWEEGR
D-1-F	Ferritin middle subunit	gi|62079570	Cellular iron ion homeostasis	Oreochromis mossambicus	106	LLSFQNKR<br>DDVALPGFSHFFK<br>DDVALPGFAHXFK
	Envelope protein	gi|3413225	Virus protein	Human immunodeficiency virus 2	50	GTNNTADITFTAPGK<br>GSDAEVAYMWTNCR<br>GSDAEVAYMWTNCRG<br>FYNLTMHCKRPGNK<br>YKGTNNTADITFTAPGK<br>TVVPITLMSGLVFHSQPINR
	Hypothetical protein A1S_1906	gi|126641951	Hypothetical protein	Acinetobacter baumannii ATCC 17978	48	ELNNMLKK<br>DDLMVRLR<br>GWVSTLENEMDSGR<br>GWVSTLENEMDSGRK<br>AEAQLGNNQILPELLR<br>MGFFWNATHQQIYR<br>GFFWNATHQQIYRELNNMLK
D-2-F	Retrotransposon protein, putative, Ty3-gypsy sub-class	gi|62734703	Putative protein, genetic elements that can amplify themselves in a genome	Oryza sativa (japonica cultivar-group)	55	ILDTMERR<br>LYTVVMAGSVTR<br>AEPAPVMVMTRR<br>EEDIPKTAFTTR<br>CRTPLMWDQVR<br>DLDAVEAQLHQLK<br>EAQKNDPDMHGLLK<br>DLEFAVDDFVYLR<br>RPYRMAANELAEVK<br>MEVDEVGGRWTFSR<br>AAHPDLFASSSESRGR<br>TTYGGNKLAELYFAR<br>MCVDYRALNEVTIK<br>IVSDRGSQFTSHFWK<br>GHCNTLNVCGIPPELNQQMEALNL<br>SIVSR
D-3-F	Retrotransposon hot spot (RHS) protein	gi|71665527	Genetic elements that can amplify themselves in a genome	Trypanosoma cruzi strain CL Brener	58	GGTDFKPKR<br>GWPYSWKEDGSTR<br>EVKEGEPPQSWTYR<br>LMVLTSDKGWPYSWK
A-1-I	Vitellogenin	gi|16151381	The protein molecule is classified as a glycolipopr	Pleuronectes platessa	87	FIELIQLLR<br>IPVEPIKAAYLPR<br>AMTKNTAPDFTSVA<br>MIQDIAVQLFMGK<br>TQNVYELQEPGVQGICK<br>KTQNVYELQEPGVQGICK
	Unknown protein	gi|23577841	Similarity to juvenile hormone sensitive hemolymph protein	Rachiplusia ou multiple nucleopolyhedrovirus	60	KINNSMFMLK<br>MSGGGNFLTLER<br>MMAFLYAEFGK<br>TTVDILEFDTR<br>GTMNAFYAPKQR<br>YLFLTSYFDLK<br>GDYESSSELKSLR<br>DNEHVPSEPMAFIR<br>FVIEENPAMSGEMSR<br>IVTRKPNDIFNEIDK<br>MSGGGNFLTLERDHFK
r-4-I	Similar to Drosophila melanogaster Prosalpha6	gi|38047741	Proteasome_alpha_type_6, regulate protein stabilization	Drosophila yakuba	43	LYQVEYAFK<br>YEAANFRYK<br>MADSRSQVQK<br>MSRGSSAGFDR<br>SGDCAVVATQKK<br>YKYGYDMPVDVLCR<br>ACSVGAKTLEANSYLEK<br>DLFLGTNEEK<br>AIVCDAPDEAK<br>EYWWNANRQEVWK<br>SSYNDDETGNYYKLR
	EMP1 variant CM5d	gi|86373996	Epithelial membrane protein 1, involved in cell proliferation,epithelial cell differentiation	Plasmodium falciparum	42	DLFLGTNEEK<br>AIVCDAPDEAK<br>EYWWNANRQEVWK<br>SSYNDDETGNYYKLR<br>QEVWKAIVCDAPDEAK

008-F	Adenine phosphoribosyl transferase	gi|116488124	Enzyme for adenine phosphoribosyl transfer	Scophthalmus maximus	58	LGVGFVLIIKK<br>EGILFRDICPILK<br>QQADILGCMVVIELK<br>GKLPGATASVAYDLEYGK<br>KQQADILGCMVVIELK
**Male and intersex**						
S-2-m	Malate dehydrogenase	gi|14583133	An enzyme that reversibly catalyzes the oxidation of malateto oxaloacetate using the reduction of NAD^+ to NADH.	Sphyraena idiastes	114	VSVPVIGGHAGK<br>KGEDFVANMK<br>EGVVECAYVR<br>TIIPLISQCTPK<br>AGAGSATLSMAYAGAR<br>ANTFVAELKGLDPAR<br>AKAGAGSATLSMAYAGAR
T-1-m	CP47	gi|21310266	More than 99% of the vertebrate ocular lens is comprised of terminally differentiated lens fiber cells. Two lens-specific intermediate filament-like proteins, the protein product of this gene (CP49 or phakinin) and filensin (also known as CP115), are expressed only after fiber cell differentiation has begun. Both proteins are found in a structurally unique cytoskeletal element that is referred to as the beaded filament (BF). Mutations in this gene have been associated with juvenile-onset, progressive cataracts and Dowling-Meara epidermolysis bullosa simplex	Cryptosporidium parvum	62	ADNSQDISLK<br>NEIFDEKSR<br>ADQGFSSPASNK<br>DLDNSERHDTK<br>TNEIQISNKEAK<br>KGLDSDMDLSPQK<br>MTNKADNSQDISLK<br>MEASSPPQNVTKMTNK<br>LSSSDLSNQSTHFTFK

Table S2. Primer sequences and PCR conditions.

Degenerate primers for swamp eel

Gene names	Primer sequences (5′-3′)	PCR conditions
Rab37	F: GTGAA RCTNCAGATCTGGGA<br>R: CTCAT RCGGGTYTCBCTGCA	95 °C, 5min; 95 °C, 30 s; 58 °C, 30 s; 72 °C, 1min, 35 cycles
Rab1A	F: GARTATGAYTATTTATTCAA<br>R: TTGTTRACATTTTCACTGGC	95 °C, 5min; 95 °C, 30 s; 58 °C, 30 s; 72 °C, 1min, 35 cycles
Pentraxin	F: ATYCTSTTCGCCTACCGMAC<br>R: GAGMACARRRTCCCACATGT	95 °C, 5min; 95 °C, 30 s; 58 °C, 30 s; 72 °C, 1min, 35 cycles
Enolase	F: TTGAKCATATCAATAAAAC<br>R: AGGATCATGAACTCYTGCAT	95 °C, 5min; 95 °C, 30 s; 58 °C, 30 s; 72 °C, 1min, 35 cycles
Rab35	F: TTGGAGACTCCAATGTGGGG<br>R: TCCCAGATCTGCAGCTTCAC	95 °C, 5min; 95 °C, 30 s; 58 °C, 30 s; 72 °C, 1min, 35 cycles
Myosin	F: AARGAGGCSTTCAACATGAT<br>R: GAGRAACATGGTRAAGTTGA	95 °C, 5min; 95 °C, 30 s; 58 °C, 30 s; 72 °C, 1min, 35 cycles
Glutathion S-Transferase M	F: GACTTCMAYGAMATGATGCC<br>R: TCCTTCATBCKYTCCACATA	95 °C, 5min; 95 °C, 30 s; 58 °C, 30 s; 72 °C, 1min, 35 cycles
Triosephosphate Isomerase B	F: CCWGCSATGATYAAGGACTG<br>R: TCTGCRATRAMYTTGGTCTG	95 °C, 5min; 95 °C, 30 s; 58 °C, 30 s; 72 °C, 1min, 35 cycles
Ferritin	F: AGYTCYCARGTGAGACAGAA<br>R: TAYTCGGCCAKGCCGTTCTG	95 °C, 5min; 95 °C, 30 s; 58 °C, 30 s; 72 °C, 1min, 35 cycles
Cbx3	F: GAGTTTYTCCTSAARTGGAA<br>R: GAGTYCTTCCACTTCQTGAG	95 °C, 5min; 95 °C, 30 s; 58 °C, 30 s; 72 °C, 1min, 35 cycles

Semi-quantitative primers for swamp eel (also used as RACE primers)

Gene names	Primer sequences (5′-3′)	PCR conditions
Rab37	F: AAAGATGGAGCTTTCCTGGC<br>R: CTCTCCATCTTCTCTCTTCA	95 °C, 5min; 95 °C, 30 s; 60 °C, 30 s; 72 °C, 30s, 28 cycles
Rab1A	F: GCTGCTCCTGATTGGTGACT<br>R: CGGTCAATCTCCTGTAGCCA	95 °C, 5min; 95 °C, 30 s; 60 °C, 30 s; 72 °C, 30s, 28 cycles
Pentraxin	F: GCCGACTATGACGAACTCAA<br>R: TCAGATCAGTCACCTCCCCT	95 °C, 5min; 95 °C, 30 s; 60 °C, 30 s; 72 °C, 30s, 28 cycles
Enolase	F: CGACAAGCTGATGCTGGACA<br>R: CATGGGAGCCTCCATTGATT	95 °C, 5min; 95 °C, 30 s; 60 °C, 30 s; 72 °C, 30s, 28 cycles
Rab35	F: TTGGAGACTCCAATGTGGGG<br>R: TGACACCGTGGGTGTTTCTG	95 °C, 5min; 95 °C, 30 s; 60 °C, 30 s; 72 °C, 30s, 28 cycles
Myosin	F: AARGAGGCSTTCAACATGAT<br>R: GAGRAACATGGTRAAGTTGA	95 °C, 5min; 95 °C, 30 s; 60 °C, 30 s; 72 °C, 30s, 28 cycles
Glutathion S-Transferase M	F: CAAGGGAGTGGTGGACTCTGATGAT<br>R: TGGAGGTCTGGAAGCGCAGGA	95 °C, 5min; 95 °C, 30 s; 60 °C, 30 s; 72 °C, 30s, 28 cycles
Triosephosphate Isomerase B	F: ACTGGGTGATCCTGGGTCACTCTG<br>R: AAGACGACCTTCTCCGTGATGCC	95 °C, 5min; 95 °C, 30 s; 60 °C, 30 s; 72 °C, 30s, 28 cycles

Ferritin	F: GGAGCTGTATGCCTCCTATG<br>R: TCTGGCTTCTTGACATCTTG	95 °C, 5min; 95 °C, 30 s; 60 °C, 30 s; 72 °C, 30s, 28 cycles
Cbx3	F: AGGCCAAGAAGGCTGAGGAG<br>R: TCTCCTCTTCTTTCTTCTCC	95 °C, 5min; 95 °C, 30 s; 60 °C, 30 s; 72 °C, 30s, 28 cycles
Hprt	F: GAACAGTGACCGCTCCATCC<br>R: TTGTCAGGGACCTCGAATCCT	95 °C, 5min; 95 °C, 30 s; 60 °C, 30 s; 72 °C, 30s, 25 cycles

Semi-quantitative primers for zebrafish:

Gene names	Primer sequences (5′-3′)	PCR conditions
Rab1A	F: AGATCTGGGATACAGCAGGG<br>R: AAGGCCTGCTCCACATTTGT	95 °C, 5min; 95 °C, 30 s; 60 °C, 30 s; 72 °C, 30s, 28 cycles
Pentraxin	F: TGACACCCGCAACGACTGAA<br>R: TTTAATCTGGTTCCCGGTGA	95 °C, 5min; 95 °C, 30 s; 60 °C, 30 s; 72 °C, 30s, 28 cycles
Enolase	F: CTTTGACTCCCGTGGAAACC<br>R: TTCTCTGCAGCACCAGCCTT	95 °C, 5min; 95 °C, 30 s; 60 °C, 30 s; 72 °C, 30s, 28 cycles
Rab35	F: ATCATCGGTGACAGCGGTGT<br>R: GCCCATCTGTTCTGCGAACT	95 °C, 5min; 95 °C, 30 s; 60 °C, 30 s; 72 °C, 30s, 28 cycles
Myosin	F: CCGCCAAAGTTTGATAAAAT<br>R: AAGTTCTCCCTATGAAGGGC	95 °C, 5min; 95 °C, 30 s; 60 °C, 30 s; 72 °C, 30s, 28 cycles
Glutathion S-Transferase M	F: CCTCTGTGCACTTTTGGCGT<br>R: CAGCAGCCGGATCTTATCCA	95 °C, 5min; 95 °C, 30 s; 60 °C, 30 s; 72 °C, 30s, 28 cycles
Triosephosphate Isomerase B	F: TTTTCGTCGGTGGAAACTGG<br>R: ACCAATGCAGGCGATCACTC	95 °C, 5min; 95 °C, 30 s; 60 °C, 30 s; 72 °C, 30s, 28 cycles
Ferritin	F: CGGCCGTTAATCGTCAGATC<br>R: ATCGCACATGTGAGGGTCGT	95 °C, 5min; 95 °C, 30 s; 60 °C, 30 s; 72 °C, 30s, 28 cycles
Cbx3	F: CAAGTCAAAGAAGGAAGTTC<br>R: TTCATCCCCCGCAATAACTA	95 °C, 5min; 95 °C, 30 s; 60 °C, 30 s; 72 °C, 30s, 28 cycles
Hprt	F: GGATGGGAACGCAAACAAAC<br>R: GGCCACTCTTTTCACCAGCA	95 °C, 5min; 95 °C, 30 s; 60 °C, 30 s; 72 °C, 30s, 25 cycles

Semi-quantitative primers for mouse:

Gene names	Primer sequences (5′-3′)	PCR conditions
Rab1A	F: AGATATGGGACACAGCAGGC<br>R: CTGTAGCTCCAGGACCCATT	95 °C, 5min; 95 °C, 30 s; 60 °C, 30 s; 72 °C, 30s, 28 cycles
Pentraxin	F: TTCACCAGCCTTCTTTCAGA<br>R: CCCAGACCGGGGCCTGATG	95 °C, 5min; 95 °C, 30 s; 60 °C, 30 s; 72 °C, 30s, 28 cycles
Enolase	F: ATTCTCAGGATCCACGCCAG<br>R: GACACTCCCAGGATGGCATT	95 °C, 5min; 95 °C, 30 s; 60 °C, 30 s; 72 °C, 30s, 28 cycles
Rab35	F: ATGGCCCGGGACTACGACCA<br>R: TTAGCAGCAGCGTTTCTTTC	95 °C, 5min; 95 °C, 30 s; 60 °C, 30 s; 72 °C, 30s, 28 cycles
Myosin	F: TCTTTGTGGATAAAAACTTC<br>R: CCAAGCTGAAGGACAGATGA	95 °C, 5min; 95 °C, 30 s; 60 °C, 30 s; 72 °C, 30s, 28 cycles
Glutathion S-Transferase M	F: CTACTACTTTAATGGCAGGG<br>R: CTGACCACCTCAACATAGGG	95 °C, 5min; 95 °C, 30 s; 60 °C, 30 s; 72 °C, 30s, 28 cycles

Triosephosphate Isomerase B	F: GGCGCCTACCAGGAAGTTCT<br>R: GAGTCCCTCTGCTAGGGCGT	95 °C, 5min; 95 °C, 30 s; 60 °C, 30 s; 72 °C, 30s, 28 cycles
Ferritin	F: CGCCAGAACTACCACCAGGA<br>R: CCAAGTGCAGTGCACACTCC	95 °C, 5min; 95 °C, 30 s; 60 °C, 30 s; 72 °C, 30s, 28 cycles
Cbx3	F: ATGGGAAAGAAACAAAATGG<br>R: ATCTCCAACATCAGAATGCC	95 °C, 5min; 95 °C, 30 s; 60 °C, 30 s; 72 °C, 30s, 28 cycles
Hprt	F: GTCCCAGCGTCGTGATTAGC<br>R: GGTCCTTTTCACCAGCAAGC	95 °C, 5min; 95 °C, 30 s; 60 °C, 30 s; 72 °C, 30s, 25 cycles

Figure S1

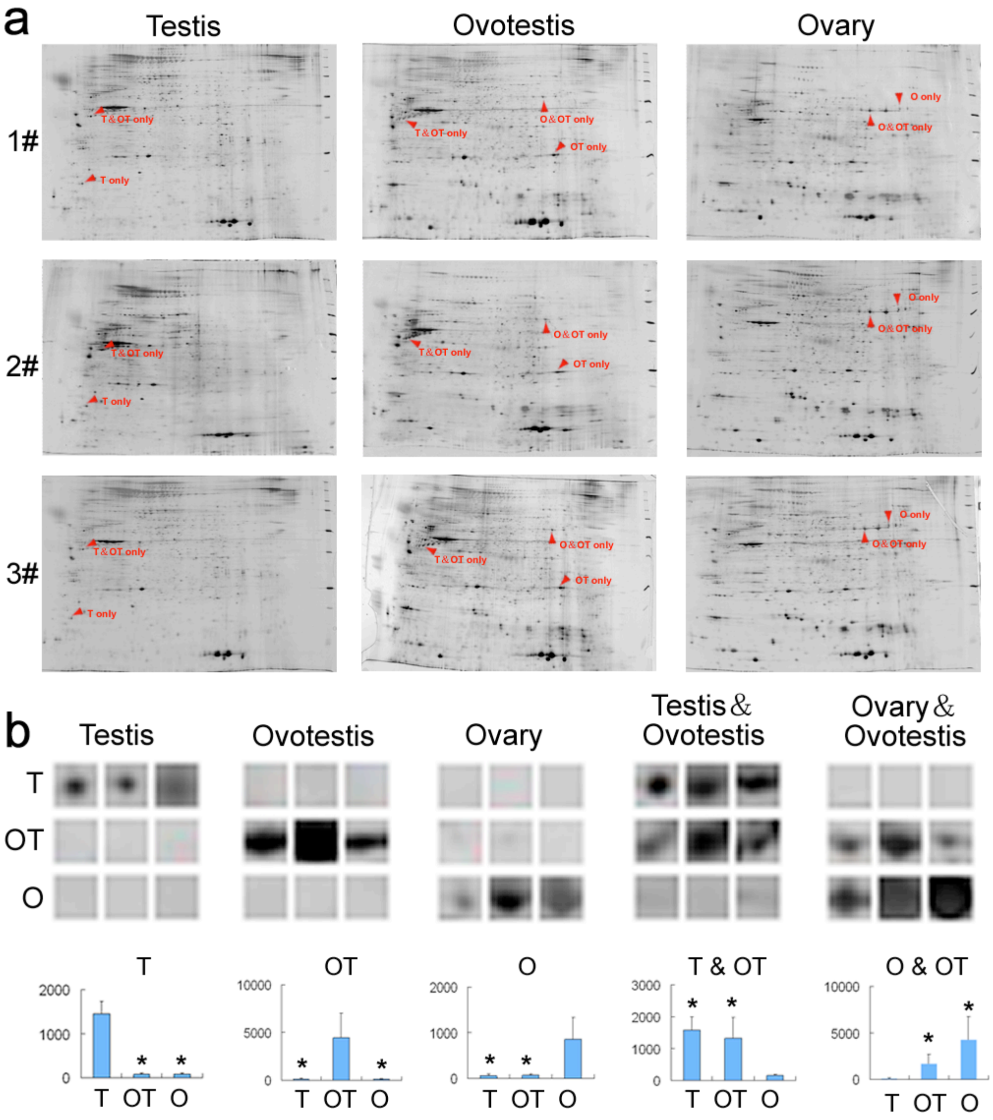

Fig. S1. Candidate spot selection

a). Representative spots for each type of gonads were labeled with red arrowheads on the images. For each type of gonads, we use three individuals to run gels respectively. T, testis, O, ovary, OT ovotestis. **b).** The intensity of each spot was calculated by ImageJ and the statistical significance of image analysis was determined by the Student's t-test (statistical level of * $p < 0.05$ is significant).

Figure S2

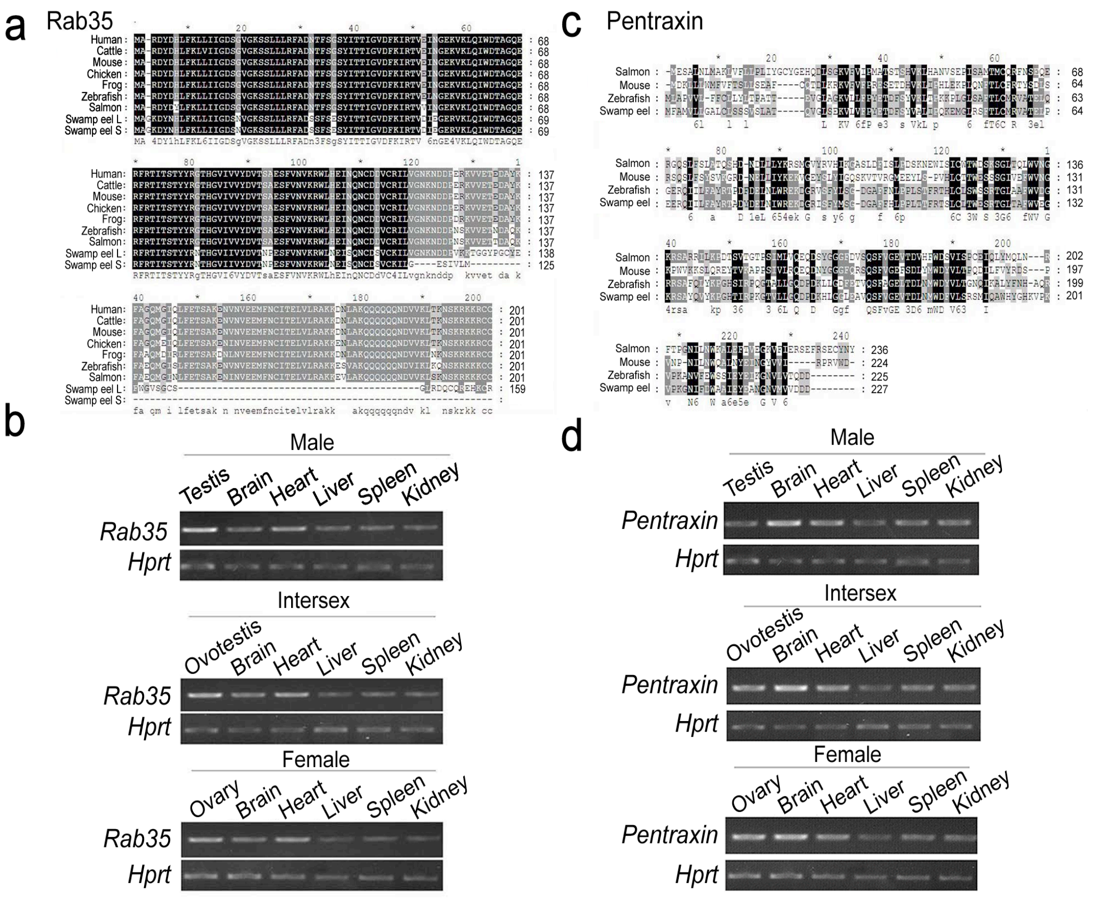

Fig. S2. Protein sequence alignments and expression of Rab35 and Pentraxin in swamp eel

a). Amino acid sequence alignments of Rab35 from human, mouse, cattle, chicken, frog, salmon, zebrafish and swamp eel. Two transcripts have been cloned in swamp eel, a longer one (L) and a shorter one (S); the main part and the 5' end are the same, only a little difference at the 3' end. **b).** RT-PCR analysis of Rab35 gene in adult tissues of each sex of swamp eel. **c).** Protein sequence alignment of Pentraxin of mouse, salmon, zebrafish and swamp eel. **d).** RT-PCR analysis of Pentraxin gene in adult tissues of each sex of swamp eel. PCR product for each gene has been run under the same experimental conditions and same sizes of gel images have been used for all genes. GenBank access numbers for swam eel are KP054390 for Rab35 isoform 1 (long) and KP054391 for Rab35 isoform 2 (short); KP054392 for Pentraxin.

Figure S3

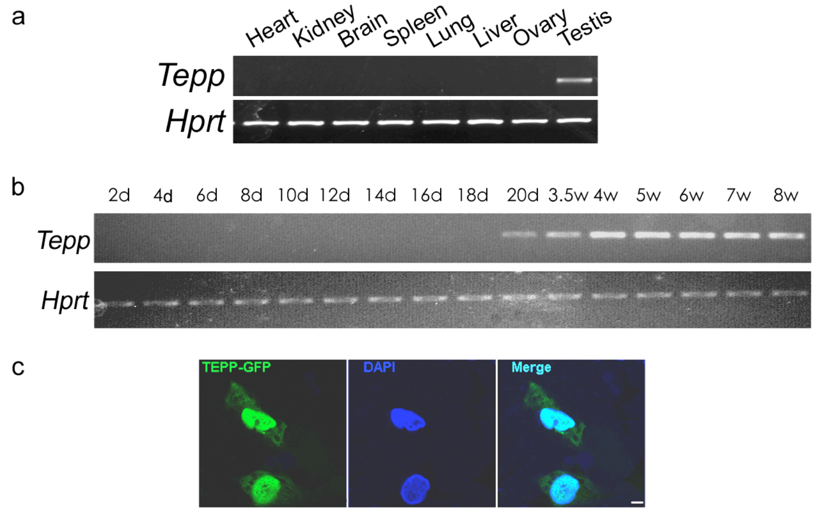

Fig. S3. Expression profile of Tepp in mouse

a). Tepp expression in adult mouse tissues. RT-PCR assay was used to detect expression of Tepp in adult mouse tissues. Tepp has a specific expression in testis, and no expression in any other tissues. **b).** Tepp expression at different stages of testis (d = days, w = week) postnatal mice. Tepp begins to express around three weeks old. PCR product for each gene has been run under the same experimental conditions and same sizes of gel images have been used for all genes. **c).** Tepp is located in th nucleus. Tepp was constructed into eGFP-n1 plasmid, and transfected in to COS7 cells. From the confocal picture, most Tepp proteins are stayed in the nucleus and little outsides. The bar, 5 μm.

Figure S4

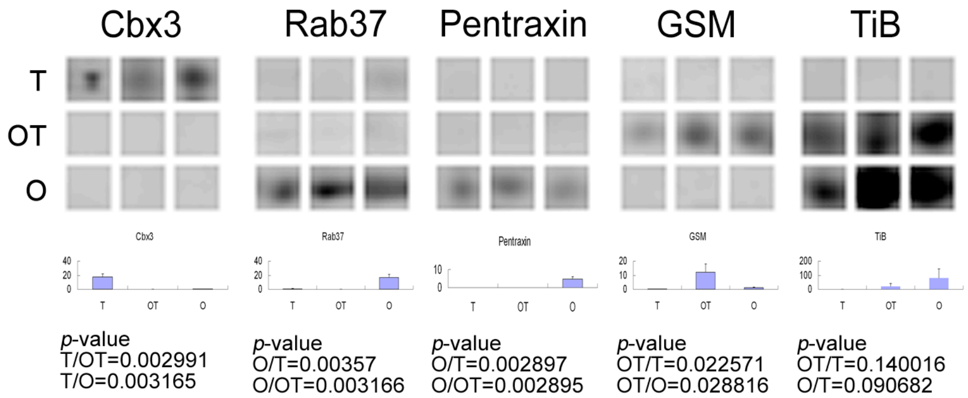

Fig. S4. Protein expression levels of three spots for each of these genes among gonad types

Three protein spots for Rab37, Cbx3, Glutathione S-transferase M (GSM), Triosephosphate isomerase B (TiB) and Pentraxin from each gonad type, related to mRNA levels in Fig. 2. The *p* values for Rab37, Cbx3, GSM and Pentraxin are shown in each panel.
